# Experiences and Needs of Core Participants in Surgical Ward Rounds: Qualitative Exploratory Study

**DOI:** 10.2196/69578

**Published:** 2025-05-15

**Authors:** Helle Poulsen, Jane Clemensen, Jette Ammentorp, Poul-Erik Kofoed, Maiken Wolderslund

**Affiliations:** 1 Department of Surgery, Lillebaelt Hospital, University Hospital of Southern Denmark, Sygehusvej 24, Kolding, DK-6000, Denmark, 45 76362665; 2 Department of Regional Health Research, Faculty of Health Sciences, University of Southern Denmark, Odense, Denmark; 3 OPEN, Open Patient data Explorative Network, Odense University Hospital, Region of Southern Denmark, Odense, Denmark; 4 Hans Christian Andersen Children’s Hospital, Odense University Hospital, Odense, Denmark; 5 Centre for Innovative Medical Technology, Odense University Hospital, Odense, Denmark; 6 Department of Clinical Research, Faculty of Health Sciences, University of Southern Denmark, Odense, Denmark; 7 Centre for Research in Patient Communication, Odense University Hospital, Odense, Denmark; 8 Department of Paediatrics and Adolescent Medicine, Lillebaelt Hospital, University Hospital of Southern Denmark, Kolding, Denmark; 9 Danish Centre for Clinical Artificial Intelligence, University of Southern Denmark and Odense University Hospital, Odense, Denmark

**Keywords:** surgical ward rounds, interdisciplinary rounds, patient participation, family involvement, digital technologies

## Abstract

**Background:**

Surgical ward rounds (SWRs) are typically led by doctors, with limited involvement from key participants, including patients, family members, and bedside nurses. Despite the potential benefits of a more collaborative and person-centered approach, efforts to engage these stakeholders remain rare.

**Objective:**

This qualitative exploratory study aims to examine the experiences and needs of doctors, nurses, patients, and their relatives during SWRs as part of a participatory design process.

**Methods:**

Data were collected through ethnographic field studies, focus groups with the health care providers, patients, and relatives, and dyadic interviews conducted as part of home visits to patients and their partners after discharge. Field notes and interview data were analyzed using systematic text condensation.

**Results:**

Lack of organization, traditional roles, and cultural norms compromised the quality, efficiency, and user experience of SWRs in multiple ways. SWRs were routine-driven, treatment-focused, and received lower priority than surgical tasks. Unpredictability resulted in unprepared participants and limited access for nurses, patients, and relatives to partake.

**Conclusions:**

The study identified a gap between the organizational and cultural frameworks governing the SWRs and the experiences and needs of key participants. Digital technologies were perceived as a potential solution to address some of these challenges.

## Introduction

A ward round is a complex hospital activity with multiple purposes and diversity in function, participants, and attendance within different hospital settings [1]. Despite its importance and global implementation, there appears to be no universally agreed-upon definition or shared understanding of a ward round [2-4]. In a literature review, Walton et al [2] identified 8 classifications, ranging from traditional rounds led by junior doctors presenting patient cases to the seniors, to interdisciplinary rounds involving health care providers from different disciplines. The primary purposes of these rounds include patient-care planning and teaching activities. Hence, ward rounds play a crucial role in ensuring person-centered care, patient safety, and high-level education [4-6]. Medical ward rounds typically involve a wide range of health care providers, including nurses and allied health care providers. Bedside interdisciplinary rounds in medical settings have been extensively investigated, showing several positive effects, such as improved interprofessional teamwork, quality of care, efficiency, and patient safety. They also promote holistic care by incorporating input from various disciplines, providing a comprehensive understanding of the patient‘s conditions and needs [7-12]. In contrast, doctors are most likely to attend and lead surgical ward rounds (SWRs) with limited involvement from other health care providers, patients, or relatives [2,13]. Logistic challenges, lack of time, and persistent traditional hierarchies may present barriers to bedside interdisciplinary rounds in surgical departments, and in some cases, contribute to the exclusion of bedside nurses [3]. A systematic review by He et al [14] identified interventions to improve SWRs, most of which involved checklists to enhance documentation and patient safety. While these checklists have demonstrated significant improvements in documentation compliance, staff understanding, and patient satisfaction, they are primarily aimed at reducing prescribing errors and critical mistakes in postoperative care, similar to practices used to improve operating room processes [5]. However, research on broader clinical and organizational frameworks to support collaborative and holistic SWRs is scarce.

Furthermore, a recent scoping review examining the use of bedside whiteboards found improvements in some aspects of patient communication in 6 of the 13 studies identified [15]. Nevertheless, the integration of these whiteboards has been insufficient to ensure significantly higher levels of patient and family participation in the SWRs [16]. As holistic and person-centered care becomes more evident in modern health care, frameworks that ensure a shared agenda during SWRs, where all relevant parties can contribute and be involved, are essential [17-19]. However, limited descriptions of the perceptions and expectations of core participants present a significant gap in understanding their roles, attitudes, and collaboration. Thus, this study aimed to investigate the experiences and needs of doctors, nurses, patients, and their relatives during SWRs.

## Methods

### Study Design

The study represents the first phase of a participatory design process, in which ethnographic methods, involving detailed observation and analysis of current practices and needs, are central [20,21]. To gain in-depth knowledge of key participants’ lived experiences and needs during SWRs, we conducted a qualitative exploratory study. Data were collected through ethnographic field studies, focus groups, and dyadic interviews conducted during home visits to patients and their partners after discharge.

The health care providers, patients, and relatives who participated in this study were also invited to serve as ambassadors in the next phase of the participatory design process, aiming to co-develop digital technologies that support a shared agenda at SWRs. Digital technologies refer to electronic systems or devices that facilitate communication, information sharing, or automation [22].

### Ethical Considerations

In accordance with the Helsinki Declaration, all participants received both written and oral information about the study’s purpose and provided informed consent. Participation was voluntary, and participants were informed they could withdraw at any time without consequence. The study was reviewed by the Regional Committees on Health Research Ethics of Southern Denmark, who determined that the project falls outside the scope of the Danish Committee Act’s definition of a reportable health science research project (S-20252000‐37) [23]. However, the study was approved by the Danish Data Protection Agency (Journal No. 20/60035), and data were stored in OPEN Analyse in compliance with the European General Data Protection Regulation [24]. Data were anonymized to ensure privacy and confidentiality. No compensation was provided to participants for their involvement in the study.

### Setting

The study was conducted at the Department of Surgery, Lillebaelt University Hospital, Denmark, from August 2021 to October 2021. The department had 26 beds and primarily treated acutely admitted adult patients with various gastrointestinal conditions, including ileus, gallstones, and pancreatitis. The length of patient admissions varied from a few days to several months for long-term stays. In 2017, Patient Care Boards (PCBs) were introduced to empower patients and their relatives to participate more actively during SWRs. Questions from the patients and an agreed-upon plan, including the names of the health care providers, dates for the next SWR, and the expected discharge, were noted on the whiteboard at the bedside.

### Participants and Recruitment

Participants in the field studies were selected through convenience sampling from those present on 3 scheduled data collection days, resulting in the inclusion of 4 doctors, 4 nurses, 16 patients, and 8 relatives willing to participate. Three observers conducted the data collection at data point 1, while 1 observer conducted the observations at data points 2 and 3. To ensure the arrival of new patients for observation, a 3-week interval between the first 2 data points as well as a 1-day interval between data points 2 and 3 were intentionally selected. This design aimed to capture a representative sample of participants over the specified time intervals. Patients and their relatives were also invited to participate in a focus group during or after admission. Initially, 14 patients and 8 relatives agreed to participate, however, 11 patients and 6 relatives later declined due to the patient’s health conditions (n=11) or transportation issues to the hospital (n=6). Consequently, the focus group included 5 participants, while 3 patients and their partners opted for dyadic interviews conducted in their own homes after discharge instead. During these interviews, patients and their partners were considered 2 separate respondents. Inclusion criteria for the study were acutely admitted, Danish-speaking patients and relatives aged 18 years or older. Participants were selected to reflect diversity in terms of sex, age, diagnosis, and length of stay (Table 1).

**Table 1.** Demographic characteristics of patients and relatives participating in focus groups and dyadic interviews.

Participants	Proportion of males, n (%)	Age (years), mean (SD; range)	Length of stay (days), mean (SD; range)
Total (n=11)	4 (36)	78.2 (8.2; 61–93)	10.0 (4.2; 7–18)
Patients (n=6)^a^	3 (50)	79.2 (5.8; 68–87)	10.7 (4.6; 7–18)
Relatives (n=5)^b^	1 (20)	77.0 (10.2; 61–93)	9.2 (3.5; 7–16)

a With a diagnosis of cholecystitis (n=2), diverticulitis (n=1), pancreatitis (n=1) and ileus (n=2)

b Partners (n=4) and adult children (n=1).

A total of 8 doctors and 5 nurses were purposively selected to participate in a focus group for the health care providers. In collaboration with the department management, a diverse group was recruited to ensure variation in sex, age, educational level, and length of experience in the ward. The term ”doctor“ will be used to refer to any doctor, regardless of seniority or position, while ”junior” and ”senior“ will indicate different levels of seniority. All nurses were registered nurses, with some holding specialized roles, such as specialist nurses or working environment representatives (Table 2). In total, 44 informants participated in the study, including participants from field studies, focus groups, and dyadic interviews.

**Table 2.** Demographic characteristics of health care providers participating in focus groups.

Participants	Proportion of males, n (%)	Age (years), mean (SD; range)	Experience (month), mean (SD; range)
Total (n=13)	6 (46)	33.7 (6.9; 25–47)	46.6 (61.1; 1–246)
Doctors (n=8)^a^	5 (63)	34.4 (6.4; 27–45)	32.0 (24.2; 1–68)
Nurses (n=5)^b^	1 (20)	32.6 (7.6; 25–47)	70.0 (88.7; 8–246)

a Junior doctors (n=5) and senior doctors (n=3)

b General nurses (n=2), specialist nurses (n=2) and working environment nurse (n=1)

### Data Collection

#### Field Studies

HP, JC, and an innovation consultant conducted 20 hours of ethnographic fieldwork by performing go-along with participants before, during, and after the SWRs. HP is an experienced nurse in the surgical specialty, though no longer involved in clinical work. JC has extensive expertise in qualitative research and participatory design, while the innovation consultant holds a Master’s degree in design management and specializes in co-operative design processes. The go-along method is a hybrid approach combining participant observation and interviewing, in which the fieldworker accompanies informants during their everyday activities, asking questions, listening, and observing to actively explore their experiences and practices as they move through and interact with their physical and social environments [25]. We found this method suitable as it enabled the observation of participants in situ while assessing their interpretations simultaneously. The fieldworkers accompanied doctors and nurses during preparations, patient room visits, and follow-up activities related to SWRs. Informal interviews were conducted to explore the transcendent and reflective aspects of the participants’ lived experiences [25]. To ensure consistency, the interviews were conducted using a set of guiding questions for the observer. These included open-ended questions such as: How did you experience the SWR? What are your needs during SWRs? Were these needs met? Additionally, more specific questions tailored to the observed situations were asked. Observations were recorded in field notes, including jottings, phrases, and additional thoughts, ideas, and questions that arose during the go-along. These jottings were expanded into detailed descriptive field notes as soon as possible [26]. Where feasible, informal interviews were audio taped and transcribed verbatim. For those not audio-recorded, comprehensive field notes were taken to ensure detailed documentation of the interviews.

#### Focus Groups and Home Visits

Focus groups were selected as a method to gain insight into the experiences and needs of participants at a group level, and to gather knowledge from the social interactions between them [27]. The format allowed each participant to elaborate on or respond to what others had shared. This process of sharing and comparing provided valuable insights into both the similarities and differences in the experiences of each group of participants [28,29]. HP facilitated the first focus group with patients and relatives, while HP and JC jointly facilitated the focus group with health care providers. Preliminary themes, identified in the field notes, were used to develop a semistructured interview guide for each focus group. The topics to discuss with patients and relatives were: preparation, timing, communication with doctors, information needs, visual explanations, role of the nurse, family participation, and digital technologies. For the health care providers, the topics were: organization, prioritizing, supervision, patient involvement, role of the nurse, family participation, visual explanations, and digital technologies. Theme cards with images were used to stimulate and structure the discussions. The focus groups each lasted 90 minutes and were held at the hospital. To supplement the data, HP conducted home visits to patients and their partners 5-16 days after discharge. During the home visits, data collection involved dyadic interviews, using the same interview guide as in the focus group with patients and relatives. In dyadic interviews, 2 participants respond to open-ended research questions through interaction [30]. This interview format allowed for the collection of in-depth, detailed data, and the interaction between the couples stimulated experiences and insights that one of the participants might not have recalled or recognized. The home visits lasted 60 minutes each, and all interviews were audiotaped and transcribed verbatim. Dot voting was used to help patients and relatives prioritize the themes they considered most important. Each participant received 5 dots and was invited to allocate them to their preferred themes, either by placing all dots on 1 theme, distributing them across multiple themes, or using a combination of these approaches.

### Data Analysis

Field notes and transcribed interview material were analyzed as a cohesive data set in an analysis matrix. The analysis followed a 4-step process guided by systematic text condensation, as outlined by Malterud [31]. First, the field notes and transcribed text were read to gain an overall impression and identify preliminary themes related to the research question. Second, meaningful units from each source were extracted to the analysis matrix and coded for classification. Third, subcategories were developed, and these were synthesized into overall categories accompanied by descriptions of the participants’ experiences. To minimize additional burden on participants, the transcripts and quotes were not shared with them for review. As a result, step 1 was solely carried out by HP. However, the preliminary themes were presented to the ambassador participants at the beginning of the next phase of the participatory design process. The participants agreed with the identified themes and did not suggest any major changes to the analysis. Nevertheless, their feedback played a crucial role in refining the final interpretation of the themes, ensuring an accurate representation of the participants’ perspectives. To ensure diverse analytical perspectives, the second step of the analysis was conducted collaboratively between HP and a research assistant. Preliminary themes, meaningful units, and codes were defined and discussed until a consensus was reached. In the first 2 steps, the data from each participant group were analyzed separately. HP and MW then defined the subcategories and synthesized them into overall categories. In these final steps, subcategories and overall categories were consolidated across all groups. The final analysis was reviewed and approved by all co-authors (Table 3). Further, a copy of the study findings was sent to the ambassador participants at the conclusion of the overall study. The Consolidated Criteria for Reporting Qualitative Studies (COREQ) were followed to promote complete and transparent reporting [32].

**Table 3.** Excerpt from the analytical process.

Step 1: Preliminary themes	Step 2: Meaningful units and codes		Step 3: Subcategories	Step 4: Overall category
	Quotes (examples)	Codes		
Prioritizing	“A doctor from the subacute track arrives and selects a patient at random from the list.” [Field note] “When we assign patients, even when we sit together, it feels somewhat random.” [Junior doctor, focus group]	The allocation of patients appears arbitrary and disorganized	Chaotic and unpredictable	Lack of organization
Organization	“Surgical ward rounds are the most unstructured I have ever encountered.” [Junior doctor, go-along interview] “There is no organization in our rounds; it’s completely chaotic, like a throwing star.” [Senior doctor, focus group]	A more deliberate organization of SWRs^a^ is required		
Supervision	“Two junior doctors arrive at 8:30 a.m. One of them asks, 'Isn’t there any adult doctor here today?” [Field note] “It feels like you’re sailing solo.” [Junior doctor, focus group]	Junior doctors face difficulty in obtaining supervision	Being unprepared	
Preparation	“You receive a long list of patients, and there’s only time to review if there’s something urgent that needs attention.” [Nurse, focus group] "If the nurses had time to review patient information, perform basic observations, calculate fluid balance, and so on before the rounds, we wouldn’t have to wait for that.” [Junior doctor, focus group]	The nurses are inadequately prepared for the SWRs		
Timing	“Suddenly, they appear, and I don’t know who they are. It takes me a moment to realize it’s a ward round.” [Patient, home visit] “They just appeared out of nowhere.” (Patient, go-along interview)	The patients are unaware of the SWRs and unprepared for them		
Role of the nurse	“The nurse discusses the patient with the doctor before the round, but does not accompany the doctor to the patient’s room.” [Field note] “The nurses you need to accompany may be occupied with another doctor.” [Junior doctor, focus group]	Often, the nurses are too busy to attend the SWRs, or the junior doctors do not invite them	Absence of nurses and relatives	
Family participation	“It’s very difficult for relatives to participate in the rounds because they span the entire day.” [Patient, focus group] “I haven’t seen a doctor at all. We were there every day, and on the first day, we waited for hours.” [Relative, home visit]	Despite waiting for hours, the relatives rarely manage to attend the SWRs		

a SWR: surgical ward round.

## Results

### Analysis

The analysis identified eight subcategories, which were consolidated into three overall categories: (1) lack of organization, (2) cultural norms, and (3) communication tools. Together, these categories offer an overview of the participants’ experiences and needs during SWRs (Textbox 1). Each category is explained in the following, supported by representative interview quotes to ensure transferability.

Textbox 1. **Subcategories and overall categories.**

**Subcategories**

- Chaotic and unpredictable
- Being unprepared
- Absence of nurses and relatives
- Routine-driven and treatment-focused
- Passive attendee roles
- Patient Care Boards
- Visual explanations
- Digital technologies

**Overall categories**

- Lack of organization
- Cultural norms
- Communication tools

### Lack of Organization

Lack of organization emerged as a dominant theme across the data, significantly compromising the quality of SWRs in several ways.

#### Chaotic and Unpredictable

All participants described the SWRs as chaotic and unpredictable. The distribution and order of patients appeared random, with little consideration for patient needs or the complexity of cases on the ward.

*I find it random which doctors are assigned to which patients, and it’s not always based on their competencies. The issue, as I see it, is that sometimes junior doctors end up with relatively complex patients. They have to consult multiple times and struggle to finalize and develop a solid plan for them.*

[Junior doctor, focus group]

Junior doctors attempted to assign patients based on their competencies, but their limited experience and knowledge hindered their ability to make appropriate selections. Both doctors and nurses expressed a need for a more deliberate patient allocation, considering patient complexity, doctor competencies, and the operational requirements of the department.

#### Being Unprepared

When patient cases were complex, junior doctors sought supervision from seniors. However, senior doctors were often preoccupied with their own tasks, making it difficult for junior doctors to receive adequate guidance. As a result, SWRs became time-consuming for junior doctors, requiring them to leave and return to patients multiple times to seek advice from seniors. Patients and their relatives noticed the varying levels of competence among the doctors and reported that inconsistent information caused confusion. All participants believed that the lack of supervision could lead to prolonged admissions, as junior doctors often delayed difficult treatment decisions. Senior doctors were generally more motivated to assess their own postoperative patients and emphasized the need for greater continuity in SWRs to better familiarize themselves with patients and conduct the rounds more efficiently. Similarly, patients expected doctors to be well-prepared and familiar with their medical histories. They noted that the lack of continuity often required them to repeat themselves. Nurses were frequently contacted by doctors at unscheduled times to participate in SWRs, which made it challenging to be adequately prepared or have in-depth knowledge of the patients. Additionally, nurses were often busy with other patients’ care or involved in other SWRs. Doctors required updated patient information from the nurses, and their preparation time was extended when the necessary data was not readily available. The lack of organization also left patients unprepared for the SWRs. They often could not distinguish between the various health care providers visiting their room and had to remain on alert for the doctor to appear at any time. As a result, they were often unaware of when the SWRs occurred and did not always recognize that they had taken place. Patients expressed a need to be notified about SWRs in advance.

*Then, suddenly, someone comes in and says, 'Hello, I’m the doctor, my name is so-and-so,' and immediately starts talking about what they know. It happens almost before you’ve fully woken up, so you can’t really listen properly… I understand they’re busy, but if I could get a little more time to (get ready), or at least have a nurse come in beforehand to let me know the doctor will be arriving shortly.*

[Patient, focus group]

Consequently, patients and their relatives expressed a desire for a shorter time window to prepare for and participate in the SWRs.

#### Absence of Nurses and Relatives

Nurses did not routinely participate in the SWRs, often due to being too busy or not being invited. While senior doctors recognized and valued their contributions, junior doctors typically preferred to conduct the rounds independently, likely due to uncertainty. Both patients and their relatives emphasized the essential role of nurses, viewing them as a crucial link between themselves and the doctors. When nurses attended SWRs, they were able to support patients by clarifying or relaying information to relatives, when needed. However, when nurses were absent, they were unable to contribute to the SWR agenda or properly follow up on prescriptions. As a result, nurses were either forced to contact the doctors later with their questions, or the doctors would reach out to update them on care plans and prescriptions. Occasionally, the nurses were not informed at all.

*Sometimes, rounds are conducted without my knowledge. I might not find out until I check the medical record at 2 PM, where it notes prescriptions from the morning, like sending a urine sample or other tasks. That gives me only an hour to fix that, and I often can’t complete everything (before shift change).*

[Nurse, focus group]

Thus, the lack of nurse attendance risks delaying the follow-up on SWRs. Nurses indicated that, if they had known the order of the rounds, they could have prioritized participation and have been better prepared with updated information about each patient. Since SWRs could last all day, relatives often waited for hours in the department yet rarely managed to attend. As a result, they felt uninformed and excluded, despite doctors and nurses generally viewing them as valuable resources for the patients. Nurses attempted to coordinate the rounds to facilitate relatives’ participation, but their success varied. Most patients felt responsible for relaying information to their relatives when they were absent during the rounds but struggled to recall the information provided. Consequently, relatives frequently turned to nurses to obtain the information they needed.

### Cultural Norms

SWRs were shaped by cultural norms that influenced participants’ roles and their ability to partake. Additionally, the rounds were defined by established routines and a narrow, treatment-focused approach.

#### Routine-Driven and Treatment-Focused

Generally, all patients were included in SWRs every day, with some undergoing unnecessary blood tests or receiving pointless rounds due to automatic processes. Nurses estimated that most patients on the ward required daily rounds, while senior doctors disagreed, arguing that direct patient interaction was not always necessary, especially when a clear treatment plan had already been established, with little or no changes needed. Most senior doctors had a treatment-oriented perspective, primarily focusing on physical symptoms. This was reflected in the patient experience, which indicated that most SWRs concentrated on specific treatments. Patients expressed that information about managing everyday life with the disease was sparse and often came too late. Likewise, nurses expressed that SWRs had a narrow focus, primarily centered on doctors presenting the treatment plan for the patient. Junior doctors were perceived as thorough in creating detailed plans but often needed guidance in prioritizing symptoms related to the immediate situation. In contrast, nurses considered their approach to be more person-centered and holistic. Compared to surgical tasks, SWRs were considered a lower priority, with senior doctors expressing a desire for them to be completed quickly.

*A real surgical department; It’s when you’re done with rounds by 9* AM *(staff laughs). Then you have time to do other things, right?*

[Senior doctor, focus group]

Patients reported that doctors and nurses were frequently interrupted during SWRs, with some leaving midconversation. Senior doctors were observed leaving the ward, either to attend to surgical tasks or to avoid distractions. They described themselves as self-directed and somewhat anarchic, acknowledging that this behavior affected the structure and organization of the SWRs. Patients and their relatives found SWRs to be very brief, with most doctors standing at the bedside. However, when doctors took the time to sit down at eye level with the patient, it not only conveyed a sense of being informed, seen, and heard but also made the patients more aware of the SWR.

*I thought it was incredible that she took the time to do that (sit down), but she did. It was as if I became myself again… Yes, I got it, this is a round…*

[Patient, home visits]

#### Passive Attendee Roles

Nurses perceived SWRs primarily as a dialogue between the doctor and the patient, adjusting their communication style to align with that of the doctor. When not invited to contribute, or if they felt the doctor was handling the situation well, they typically refrained from speaking out. As a result, when nurses accompany doctors to the patient room, they often adopt a passive, listening role. Similarly, relatives who were able to attend SWRs were generally not actively engaged in the conversation. The time-constrained behavior of doctors, combined with a sense of deference to authority, limited knowledge, and the unpredictability of the rounds, often prevented patients and relatives from asking questions. Allowing them the opportunity to prepare by noting questions in advance could help alleviate this hesitation.

*If we knew we could speak with the doctor, say at 11 AM, my daughter and I would definitely have prepared. We would have written down a whole list of questions…*

[Relative, home visits]

Scheduled SWRs with a clear agenda would help patients and relatives to prepare in advance and feel more confident in asking questions.

### Communication Tools

Participants explored various communication tools as potential solutions to address their needs and the challenges encountered during SWRs.

#### Patient Care Boards (PCB)

Patients and relatives expressed a need for clearer information about care plans and saw the PCB as a useful tool for staying informed. However, they often found it inadequately updated. Some nurses used the PCB before SWRs to identify questions that patients might have for the doctor. While doctors recognized the value of the PCB in aligning expectations and keeping patients informed, they generally preferred that the nurses took responsibility for updating it.

#### Visual Explanations

Some doctors used visual aids, such as drawings of the gestational system or x-rays, to explain the disease, examinations, or treatments offered to the patients. Most patients and their relatives reported that this approach enhanced their understanding.

*We don’t know what’s happening beneath the surface of the skin… A picture would make everything clearer, as I could immediately identify where the stoma is located, which would help me understand the source of the pain.*

[Relative, home visits]

#### Digital Technologies

Patients and relatives saw potential in using digital technologies, such as apps for information or video communication with relatives. They discussed the use of these technologies by combining theme cards they felt were related to one another.

*If you group these together (points to three theme cards)... it makes a difference, both in terms of the timing of the rounds and the involvement of relatives, if digital technologies could be used.*

[Patient, focus group]

Patients and relatives believed that digital technologies could help them engage more actively by providing better access to information about the timing of the SWRs and improving their ability to prepare and attend. However, they noted that older individuals often lack digital competencies and would require guidance or alternative options. While nurses were generally supportive of digital technologies, most doctors viewed them as irrelevant or disruptive. Patients emphasized that while digital technologies could facilitate communication, human interaction, and personal presence remained their top priority.

## Discussion

### Principal Results

Through our investigation of the experiences and needs of core participants in SWRs, we identified several factors that compromise the quality, efficiency, and overall experience of these rounds. The most significant factors were a lack of organization and the low priority given to the SWRs compared to surgical tasks. Combined with a routine-driven and treatment-oriented focus, along with the influence of cultural and hierarchical norms, these issues create a snowball effect resulting in unpredictability, unprepared participants, and limited opportunities for nurses, patients, and relatives to partake. Assigning a dedicated coordinator to ensure that all participants are informed of the what, when, where, and who of each round will ensure that each team member is invited and leaves with clear takeaways. Further, specific objectives and time frames for each round will help maintain focus and prevent them from extending throughout the day. Patients and their relatives recognized the potential of using digital technologies to enhance their engagement in SWRs. While nurses supported the use of technologies to ensure broader participation, doctors, however, were skeptical about their practical applicability. As highlighted in a feasibility study by Johannink et al [33], medical students preferred face-to-face interactions over digital formats like video-transmitted SWRs. This finding aligns with the perspectives shared by the participants in our study, emphasizing that, while digital tools can assist in enhancing communication, they cannot replace the essential in-person care and interaction required in clinical settings.

### Comparison With Prior Work

The low priority given to SWRs is a widely recognized issue. Savage et al [3] and Shetty et al [34] noted that SWRs are commonly perceived by senior doctors as a short activity and they seldom take precedence over other surgical responsibilities. In their study on team dynamics, Bonaconsa et al [13] highlighted the significant pressure placed on seniors due to their numerous competing commitments and informal queries throughout the day. As a result, the organizational structure of surgical departments limits the availability of senior doctors on the wards. Consequently, junior doctors play a crucial role in conducting SWRs, often learning through hands-on experience or by emulating their senior colleagues [4,6,35-38]. In line with our findings, Monash et al [39] reported that senior doctors generally hold positive attitudes toward interdisciplinary rounds with nurses. However, junior doctors expressed lower satisfaction, perceiving them as more time-consuming. The feasibility of interdisciplinary rounds was therefore positively influenced by the presence of senior doctors. In our study, lack of organization led to nurses often not participating in SWRs, a finding consistent with other studies that identify differing work routines as a major barrier to nurse involvement [4,40-42]. Observational studies further support this issue, showing nurse attendance at SWRs ranging from only 13% to 44% [3,38,41,43]. Interdisciplinary rounds have been shown to decrease mortality rates, reduce hospital stays, and lower health care costs [41]. Such collaboration ensures that all team members, including nurses, patients, and relatives, are prepared and have access to participate meaningfully in SWRs. The lack of organization left nurses in our study unprepared, requiring doctors to spend additional time gathering relevant patient data. Moreover, the absence of nurses during SWRs resulted in gaps in the handover of care plans and delays in follow-up. Consistent with this, Bonaconsa et al [13] found that prescriptions not directly communicated to nurses could delay follow-up by as much as a day. Furthermore, several studies indicate that when nurses attend SWRs, the number of inquiries and calls to doctors later in the day is reduced [7-9,44]. Prioritizing SWRs by allocating dedicated time for them would allow nurses to plan their day effectively, ensuring they are prepared and able to participate. Further, a facilitator might break down malignant power hierarchies and guide the rounds by determining which team members should be involved.

The lack of organization in SWRs is a well-documented challenge for patients and their relatives as well. Swenne et al [45] found that the timing of SWRs varied from day to day. Additionally, Schwartz et al [7] identified several logistical barriers to patient participation, such as patients not being present, sleeping, or lacking interpreter assistance. Despite these challenges, some patients in our study took proactive steps to prepare by noting questions well in advance, often with the support of nurses using the PCB. Walton et al [46] found that patients familiar with the health care system often learn to navigate the SWR process to ensure their needs are met. These patients prepare by considering both the information they need to provide and the questions the doctor may ask. Several studies suggest that adopting a structured approach with a fixed starting time optimizes the use of patients’ time, allows them to be better prepared and actively participate, and makes it easier for family members to attend [4,45-47]. Relatives in our study rarely managed to attend the SWRs, a finding consistent with previous research [16], which reported a low relative attendance rate of just 19%. Studies suggest that the presence of relatives enhances communication between doctors and patients, with relatives noting that being present allows them to participate in decision-making [47,48]. In our study, both doctors and nurses acknowledged relatives as valuable resources, but the lack of organization hindered their attendance. However, providing relatives with clear explanations and valuable information during the SWRs can reduce the need for additional meetings outside of rounds [48]. Similarly, we observed that relatives often sought the nurses between rounds to obtain the information they needed. Research highlights the essential role nurses play in ensuring patients fully understand the information provided, bridging the gap between doctors and relatives [4,45]. When nurses were absent from SWRs, the responsibility shifted more heavily to the patients. As a result, many patients in our study felt obligated to relay information to their relatives when neither they nor the nurse were present, yet they often struggled to recall the information given. Coordinating SWRs through digital technologies to connect relatives to the bedside, either physically or digitally, might enhance the overall experience and improve the efficiency of family involvement.

Another crucial aspect is the influence of cultural and hierarchical norms on participants’ ability to engage. Studies have shown that nurses often perceive SWRs as primarily belonging to doctors, leading to hesitance in voicing their concerns, even when such omissions could compromise patient safety [3,49]. In our study, we observed nurses adapting their communication style to align with that of the doctors, typically refraining from interrupting. However, when doctors actively involve nurses in SWRs, it fosters more comprehensive discussions about patient or family concerns [50]. Recognizing and valuing nursing input in SWRs is, therefore, essential for improving the focus and quality of these rounds. Patients frequently expressed difficulty distinguishing between the numerous health care providers visiting their rooms. Similarly, Swenne et al [45] found that patients struggled to identify names and professions, with small nametags providing little assistance. Observational studies reveal inconsistent self-introduction practices among health care providers, with rates ranging from 81% to as low as 15% [46,51,52]. Furthermore, our findings revealed that patients perceived SWRs as brief, disruptive, and overly focused on medical issues. Descriptive studies show that the average time spent at the bedside ranges from 7.5 minutes during medical ward rounds to as little as 2.3 minutes during SWRs [34,43,50,53]. Similarly, several studies report that the short duration, frequent interruptions, and emphasis on medical decision-making hinder patients from engaging in a meaningful way [4,45,46,51,52,54]. In contrast, Ratelle et al [55] found no correlation between the duration of the SWR and patient experience, suggesting that the quality of time spent at the bedside is more important. Similarly, Iversen et al [56] discovered that person-centered communication did not affect the length of consultations. In ward rounds, patients emphasize the importance of active listening skills, body language, and the doctor’s physical positioning [55]. Consistent with these findings, patients in our study valued when doctors sat at eye level with them, underscoring that human interaction and presence were paramount. Video filming the rounds for training purposes might offer valuable insights [33]. Such recordings could facilitate self-reflection and team feedback, as well as help identify opportunities for further improvement in the structure and effectiveness of future rounds.

### Limitations

We successfully recruited a diverse group of health care providers, with variations in sex, age, experience, and education. However, we observed a significant dropout among patients and their relatives, highlighting the challenges of engaging this vulnerable and hard-to-reach group. Furthermore, the majority of relatives in our study were women, with female partners comprising the majority. This aligns with previous studies, which have found that most relatives participating in SWRs are female [16]. As a result, we lack insights into the experiences and needs of male relatives, as well as an understanding of the reasons for their absence. Involving our participants in the very early stages of the study could have provided valuable insights and adjustments to optimize our study design and recruitment process, making it more suitable for our target group. However, we remained adaptable throughout the recruitment process and conducted the home visits, which allowed us to recruit a broader range of patients and enhance the diversity of our sample. Furthermore, the home visits yielded more nuanced data, as the dyadic interview format allowed for in-depth explanations and follow-up questions, providing a richer understanding of the experiences of both patients and their relatives.

The single-center design of our study may limit the generalizability of our findings, as the specific department may have unique workflows and a distinct round culture. However, the alignment of our results with existing literature strengthens the reliability and consistency of our findings. To mitigate the influence of unacknowledged preconceptions of the research team, a diverse group of researchers with varying experiences and expertise conducted the data collection and analysis. This collaborative approach was intended to enhance the credibility and rigor of the study. All authors emphasized maintaining openness to the participants’ lived experiences, presenting the data as they emerged rather than allowing personal or theoretical frameworks to shape or interpret the findings. However, our background in participatory design naturally drew our focus toward digital technologies as potential solutions to meet user needs, which we sought to explore through our informants. We chose to analyze the diverse experiences of participants as a single entity, which may have limited the depth and nuances of the results. However, in order to develop high-quality, user-centered SWRs that address the needs of all core participants, we aimed to explore the complexity of experiences and needs in their entirety.

### Conclusions

This study highlighted a significant gap between the organizational and cultural frameworks governing the SWRs and the experiences and needs of key participants. To bridge this gap, it is essential to address the lack of organization, prioritization, and timing of the SWRs. Patients and their relatives recognized the potential of using digital technologies to address some of these challenges. However, due to the skepticism toward technology among doctors and the low priority given to SWRs, it is crucial to involve them in developing these technologies. Nurses, on the other hand, expressed support for using digital technologies to enhance broader participation. Therefore, the next phase of this research should focus on co-developing digital technologies that facilitate more structured SWRs, fostering active involvement from all key participants. This approach aims to ensure successful implementation while improving the overall quality, efficiency, and user experience.

## Supplementary material

10.2196/69578 Checklist 1 Consolidated Criteria for Reporting Qualitative Studies (COREQ) Checklist.

## Abbreviations

COREQ: Consolidated Criteria for Reporting Qualitative Studies
PCB: Patient Care Board
SWR: surgical ward round
